# Effects of Ceftiofur and Chlortetracycline Treatment Strategies on Antimicrobial Susceptibility and on *tet*(A), *tet*(B), and *bla*_CMY-2_ Resistance Genes among *E. coli* Isolated from the Feces of Feedlot Cattle

**DOI:** 10.1371/journal.pone.0080575

**Published:** 2013-11-19

**Authors:** Neena Kanwar, H. Morgan Scott, Bo Norby, Guy H. Loneragan, Javier Vinasco, Matthew McGowan, Jennifer L. Cottell, Muckatira M. Chengappa, Jianfa Bai, Patrick Boerlin

**Affiliations:** 1 Department of Diagnostic Medicine/Pathobiology, College of Veterinary Medicine, Kansas State University, Manhattan, Kansas, United States of America; 2 Department of Large Animal Clinical Sciences, College of Veterinary Medicine, Michigan State University, East Lansing, Michigan, United States of America; 3 International Center for Food Industry Excellence, Department of Animal and Food Sciences, College of Agriculture and Natural resources, Texas Tech University, Lubbock, Texas, United States of America; 4 Department of Pathobiology, Ontario Veterinary College, University of Guelph, Guelph, Ontario, Canada; Rockefeller University, United States of America

## Abstract

A randomized controlled field trial was conducted to evaluate the effects of two sets of treatment strategies on ceftiofur and tetracycline resistance in feedlot cattle. The strategies consisted of ceftiofur crystalline-free acid (CCFA) administered to either one or all of the steers within a pen, followed by feeding or not feeding a therapeutic dose of chlortetracycline (CTC). Eighty-eight steers were randomly allocated to eight pens of 11 steers each. Both treatment regimens were randomly assigned to the pens in a two-way full factorial design. Non-type-specific (NTS) *E. coli* (n = 1,050) were isolated from fecal samples gathered on Days 0, 4, 12, and 26. Antimicrobial susceptibility profiles were determined using a microbroth dilution technique. PCR was used to detect *tet*(A), *tet*(B), and *bla*_CMY-2_ genes within each isolate. Chlortetracycline administration greatly exacerbated the already increased levels of both phenotypic and genotypic ceftiofur resistance conferred by prior CCFA treatment (*P*<0.05). The four treatment regimens also influenced the phenotypic multidrug resistance count of NTS *E. coli* populations. Chlortetracycline treatment alone was associated with an increased probability of selecting isolates that harbored *tet*(B) versus *tet*(A) (*P*<0.05); meanwhile, there was an inverse association between finding *tet*(A) versus *tet*(B) genes for any given regimen (*P*<0.05). The presence of a *tet*(A) gene was associated with an isolate exhibiting reduced phenotypic susceptibility to a higher median number of antimicrobials (n = 289, median = 6; 95% CI = 4–8) compared with the *tet*(B) gene (n = 208, median = 3; 95% CI = 3–4). Results indicate that CTC can exacerbate ceftiofur resistance following CCFA therapy and therefore should be avoided, especially when considering their use in sequence. Further studies are required to establish the animal-level effects of co-housing antimicrobial-treated and non-treated animals together.

## Introduction

Antimicrobial resistance is of global public health concern because it can exert enormous clinical and financial burdens on health care systems worldwide [1], [2]. Antimicrobials are widely used in animal agriculture as therapeutic, prevention, control, and growth promotion agents [3]. Although not without controversy, several reports have indicated that antimicrobial use in food animals has been associated with subsequent development of resistance to antimicrobials in bacterial pathogens from humans [4], [5], [6]. Various intervention strategies have been proposed or established by regulatory organizations around the world in an attempt to address this problem. Such strategies include: 1) banning of antibiotics as agricultural growth promoters [7], [8], 2) removing certain antibiotic classes from the market, and 3) recommending that some classes of antibiotics never be approved for food animal use [9]. An outright ban or removal of antimicrobials is speculated to eventually result in reduced antimicrobial resistance among bacteria, but such actions may also impede veterinarians' or producers' ability to prevent, control, and treat diseases; paradoxically, this could actually increase public health risk [10], [11]. The need is urgent to better understand factors that contribute to the dissemination, propagation, and persistence of antimicrobial resistance determinants among both commensal and pathogenic enteric bacteria and to design treatment strategies at the animal, pen, and farm levels to control and mitigate this global problem [12].

Ceftiofur, a third generation cephalosporin, belongs to the same general class of antibiotics as ceftriaxone and is classified as a critically important antibiotic by the World Health Organization [13]. Ceftriaxone is highly valued in human medicine, especially for treating invasive salmonellosis in children [14], [15]. Resistance to ceftiofur is regarded as problematic because shared resistance determinants may confer resistance to ceftriaxone. This paper describes a study designed to evaluate the effects of two different sets of treatment strategies on phenotypic and genotypic ceftiofur resistance among non-type-specific (NTS) *Escherichia coli* isolates in feedlot cattle. The first set of treatment strategies was to evaluate the differential effect of whole-pen versus individual-animal level ceftiofur treatment (ceftiofur crystalline-free acid: CCFA, a long-acting ceftiofur formulation). The whole-pen treatments with CCFA were meant to mimic a ‘metaphylaxis’ or ‘control’ label use such as for a bovine respiratory disease (BRD) outbreak in feeder cattle. On the other hand, single individual-animal treatment in a pen of otherwise untreated and healthy cattle were meant to mimic the sporadic treatment of BRD cases. Among pens in which only a single animal received the CCFA therapy, the remaining animals were expected to serve as a ready source of more susceptible enteric bacteria, which could help repopulate the gut flora of treated cattle. Agreement is far from unanimous, but many scientists accept that antibiotic-resistant bacteria carry resistance genes at a relative fitness cost [16], [17]. Readily available susceptible bacteria — bacteria devoid of the resistance gene — may help promote rapid re-colonization of the host gut (treated animal) by outcompeting resistant bacteria that tend to dominate post-treatment periods [18]. This study exploited these principles to determine if re-colonization was affected by higher levels of exposure to susceptible bacteria, or even to those bacteria resistant to other antimicrobials.

The second set of treatment strategies was either to feed or not feed chlortetracycline (CTC) at therapeutic doses following CCFA treatment. Our previous work demonstrated that CTC resulted in a temporary decrease in the prevalence of ceftiofur resistant *E. coli*, especially while it was being administered in the feed [19]. Chlortetracycline treatment without prior CCFA administration in the study by Platt *et al*. (2008) appeared to differentially favor *E. coli* isolates that were singly resistant to tetracycline versus those that exhibited both ceftiofur and tetracycline resistance. Those earlier results suggested that CTC might help minimize the proliferation and accumulation of ceftiofur resistant bacteria in animal agriculture settings. Our second treatment strategy was designed based on those results. We hypothesized that CTC would expedite the return of ceftiofur resistance to baseline levels among non-type-specific (NTS) *E. coli*, whether following metaphylaxis or individual therapy indications. Our focus was on pen-level interpretations, both for treatments and for outcomes. This is consistent with aiming to reduce the overall carriage of resistant bacteria in truckloads of cattle shipped to slaughter.

The effects of these two sets of treatment strategies (i.e., differential CCFA treatment and subsequent CTC administration) were determined by examining the susceptibility profiles and the differential selection and co-selection of ceftiofur and tetracycline resistance genes among NTS *E. coli* isolates from cattle feces. Further, associations of the resistance genes among themselves and with the various phenotypic multidrug resistant (MDR) counts were evaluated. The phenotypic MDR counts in this study were defined as the number of antimicrobials (present on a single 15-drug panel) toward which individual isolates exhibit phenotypic resistance. These observations were explored to better explain the phenomena observed in the current study as well as the earlier trial by Platt *et al.* [19]. This approach aided in understanding of the factors contributing to the development and accumulation of MDR determinants.

## Methods

### Study Design

A 26-day randomized controlled trial was conducted at a research feedlot located at West Texas A&M University in Canyon, TX. All procedures used were reviewed and approved by the Amarillo-Area Cooperative Research, Education, and Extension Triangle Animal Care and Use Committee (Protocol No. 2008-07), and by the Clinical Research Review Committee at Texas A&M University (CRRC # 09-35).

Eighty-eight steers were allocated to eight pens of 11 steers each, such that average pen steer weights were similar. The two pen-level treatment strategies were randomly assigned to these eight pens in a complete two-way full factorial design resulting in four different treatment groups. Factor 1 determined whether all 11 animals in a pen versus 1 out of 11 animals were treated with CCFA. Factor 2 was a follow-up CTC regimen referring to cattle in pens receiving CTC in feed following the CCFA regimens. A third factor (not randomized) in the statistical models was the effect of day of study (period) on the level of antimicrobial resistance measured in each of the phenotypic and genotypic endpoints. The drugs, dosages, routes of administration, and treatment regimens are presented in Table 1.

**Table 1:** Drugs, dosages, routes of administration, and treatment regimens.

Drug Name	Brand Name	Dose (by BW)	Route of administration	Treatment regimen(s)	Days in regimen
Ceftiofur crystalline-free acid (CCFA)	Excede®	6.6 mg/kg	Subcutaneous, base of ear	1 (Day 0)	1
Chlortetracycline (CTC)	Aureomycin®	22 mg/kg	Top-dressed on feed	3 (Days 4–8,10–14,16–20)	5

BW = body weight

Excede®, (Zoetis Animal Health, Florham Park, NJ)

Aureomycin®, chlortetracycline complex equivalent to 220.5 g of chlortetracycline/kg of premix (Alpharma, Bridgewater, NJ)

**Figure 1:**
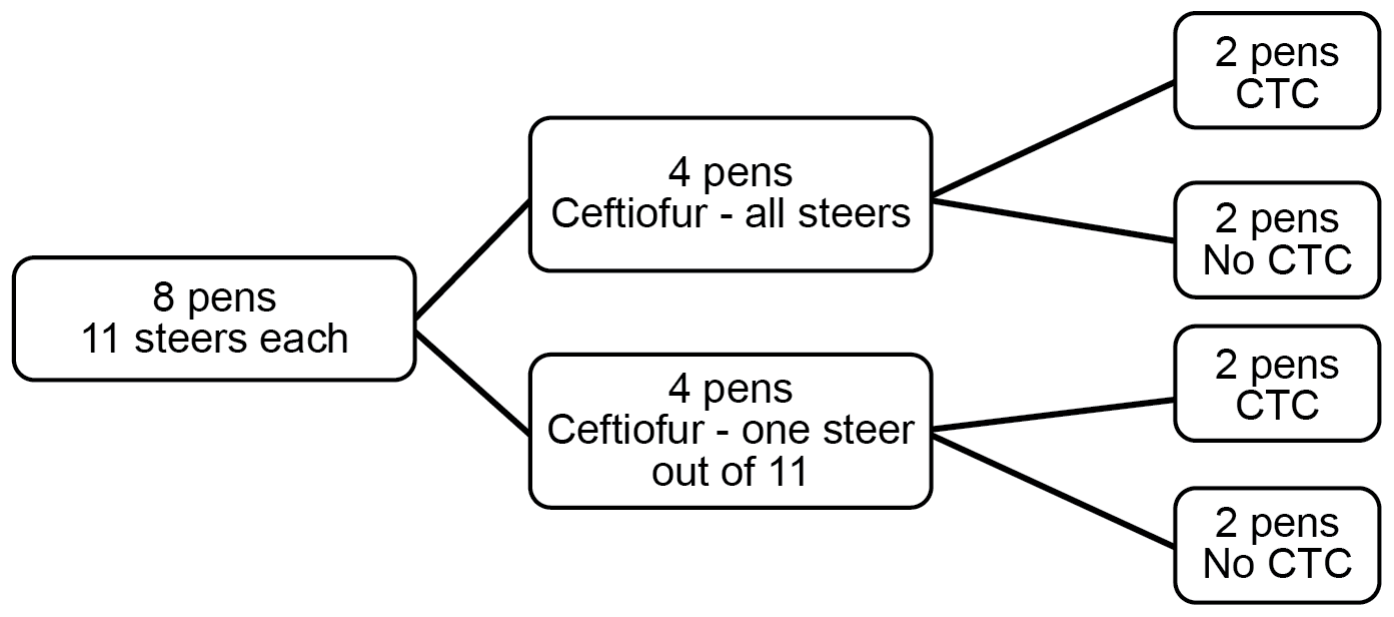
Schematic diagram of the study design. The two sets of treatment strategies were assigned in a two-way full-factorial manner. Number of pens assigned to each treatment and number of animals within each pen are shown above.

All steers in four out of eight pens were given CCFA treatment (Excede®, Zoetis Animal Health, Florham Park, NJ) as a single-dose regimen of 6.6 mg/kg administered subcutaneously at the base of the ear) on Day 0. This is the recommended labeled dose and route of administration for treatment of bovine respiratory disease (BRD) and bovine foot rot and for control of BRD. Two of these pens each received three separate 5-day regimens (with a 1-day break in between) of 22 mg/kg CTC (Aureomycin®, chlortetracycline complex equivalent to 220.5 g of chlortetracycline/kg, Alpharma, Bridgewater, NJ) *via* top-dressing after the morning feed was delivered according to the label directions and starting at Day 4. A therapeutic labeled dose of CTC recommended for the control and treatment of bacterial conditions, such as pneumonia caused by *Pasteurella multocida*, was used. The three consecutive 5-day treatment regimens were administered in a similar manner to our previous study [19] to aid inter-study comparisons. The current study was designed to evaluate the potential role of CTC as an intervention strategy to control ceftiofur resistance. Peak levels of ceftiofur resistance were expected to occur on or about Day 6 post CCFA treatment [20]; therefore, CTC was administered subsequent to ceftiofur treatment starting on Day 4. Chlortetracycline administration in this study was expected to control the increased ceftiofur resistance caused by CCFA treatment. In the remaining four pens, CCFA was administered to only 1 out of 11 steers within the pen. In two of these pens, CTC was likewise given to all animals on the same schedule and dosing regimen as described above (Figure 1).

### Sample collection

Steers were restrained in a squeeze chute at 06:00 every other day. Fecal grab samples of approximately 50 g were collected per rectum with a new obstetric sleeve glove and placed in individual plastic cups. All samples were transported on ice to the laboratory on the day of sample collection. Fecal samples were mixed with glycerol at a 1∶1 ratio; 4 ml of the mixture was added to 5 ml cryo-vials and stored at −70°C for further bacterial culture and isolation as well as phenotypic and genotypic analysis of same. Two fecal samples were not collected due to the death of a single steer late in the study period.

### Isolation of non-type-specific E. coli

A microbiological culture-based method was used for NTS *E. coli* isolation from the glycerol-preserved frozen samples. The procedures for NTS *E. coli* isolation and antimicrobial susceptibility testing were adapted from previous work [19]. Briefly, 200 milligrams of fecal sample was mixed with 1.8 milliliter of buffered peptone water, and the suspension was streaked onto MacConkey agar (BD Difco™, Sparks, MD). Plates were incubated at 37° for 18–24 hours. Three separate and distinct colonies (slightly convex, magenta-colored colonies surrounded by a dark pink area) were streaked on three separate MacConkey plates and incubated for 18–24 hours. This step was added to the protocol to ensure we obtained pure cultures of NTS *E. coli*.

We performed a quality control experiment to test if this single extra passage would lead to the loss of plasmid/resistant determinants and thereby cause significant differences insusceptibility results. The head-to-head experiment was conducted on 33 *E. coli* isolates derived from Day 4 fecal samples. These samples were obtained from three pens in which all animals received CCFA treatment. *E. coli* isolates arising from these fecal samples were most likely to harbor the ceftiofur resistance genes, which have been shown to be almost exclusively plasmid borne in North America [21], [22], [23]. The results revealed an extremely high level of agreement (median κ = 0.93) between the susceptibility results obtained from either a single, or double passage for all 15 antimicrobials. The paired t-test, comparing the MIC values and testing resistant proportions between the two groups, also revealed no significant differences between the results obtained from the two passage approaches (p>0.05). Therefore, we concluded that an extra passage did not lead to significant differences in antimicrobial susceptibility results, but it aided in ensuring we had pure cultures to perform further phenotypic and genotypic analyses. An indole spot test was performed on each isolate. Although not definitive for *E. coli*, when combined with the prior probabilities that arise from the selective medium and the morphological selection (including lactose fermentation) the post-test probabilities are well in excess of 99% [20]. Previous work [20] has suggested that little is gained from biochemical confirmation of NTS *E. coli* isolates over simple morphological selection of a typical colony obtained from MacConkey agar; in that study, biochemical assays confirmed 99.9% of the typical colonies on MacConkey agar to be *E. coli*. A single colony from each of the three MacConkey plates was streaked onto three separate Tryptic soy agar plates (BD Difco™, Sparks, MD) and was incubated for 18–24 hours. The NTS *E. coli* isolates from the TSA plates were further used for the antibiotic susceptibility testing.

DNA from NTS *E. coli* isolates was extracted for genotypic analysis by suspending a colony in 500 µl of nuclease-free water (Qiagen, Valencia, CA) and then heating the suspension at 95°C for 10 minutes. The NTS *E. coli* DNA samples were stored at −20°C for further qualitative detection of *tet*(A), *tet*(B), and *bla*_CMY-2_ genes. The plasmids encoding *bla*_CMY-2_ gene is predominantly associated with ceftiofur resistance from both humans and animals isolates in the United States [24], [25], [26]. Therefore, the *bla*_CMY-2_ gene was chosen to predict the genotypic ceftiofur resistance among the isolates. The two tetracycline genes [*tet*(A) and *tet*(B)] were chosen because they have been reported to be the most abundant tetracycline resistance genes detected among *E. coli* in cattle in the United States [27].

### Antimicrobial susceptibility testing

Fecal samples from all animals in all eight pens representing all four treatment combinations on Days 0, 4, 12, and 26 were analyzed. Three NTS *E. coli* isolates from a total of 350 fecal samples each (1,050 NTS *E. coli* isolates) were tested for their antimicrobial susceptibility profile and gene presence.

**Figure 2:**
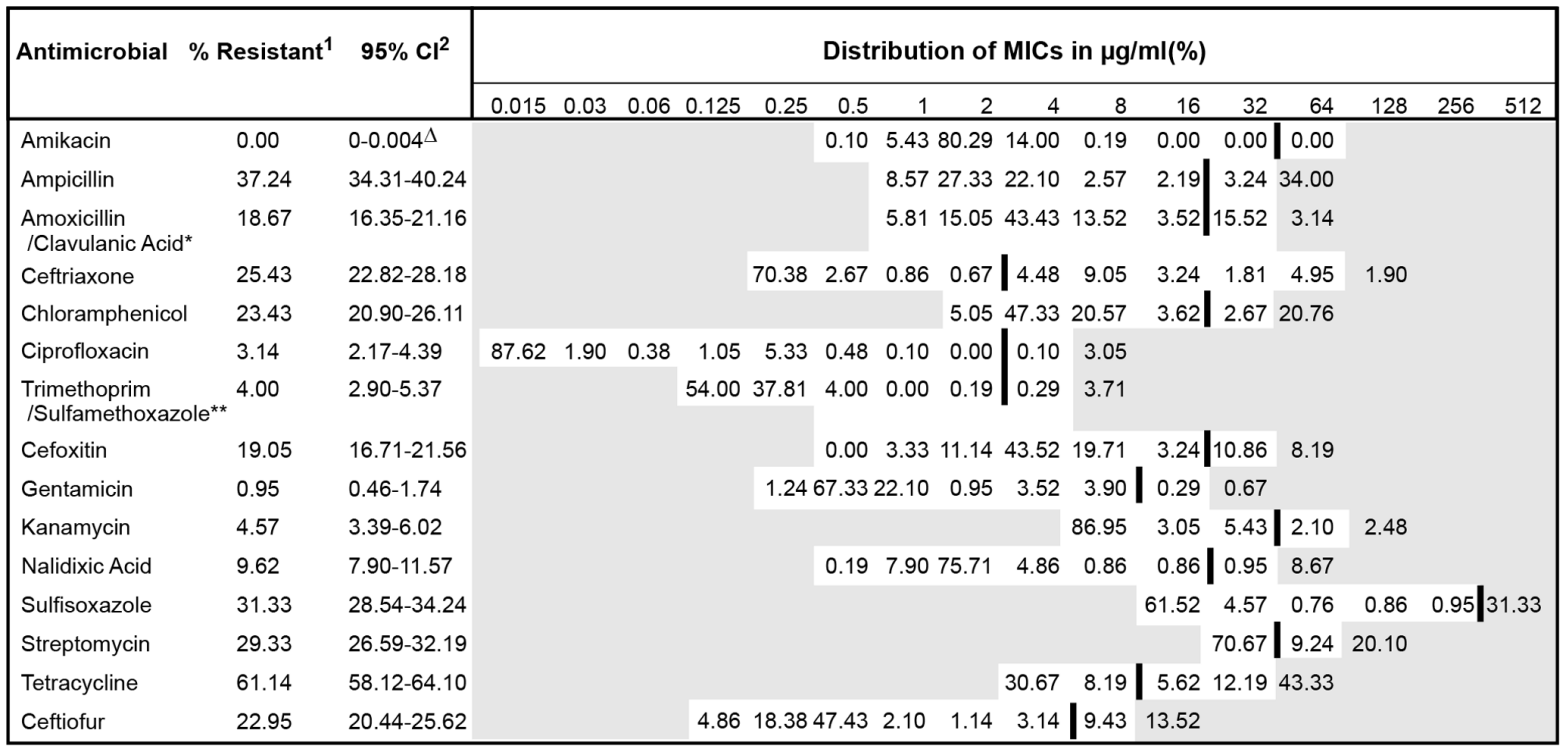
Distributions of minimum inhibitory concentrations of 1,050 non-type-specific *E. coli* isolates against 15 antibiotics. Unshaded areas indicate the dilution range of the Sensititre® plate used to test isolates. Vertical bars indicate the CLSI resistance breakpoint when available, or else NARMS consensus breakpoint. Sum of numbers beyond vertical bar represents the percentage of isolates that grew beyond the CLSI breakpoint (or, NARMS consensus breakpoint). These were considered resistant in this study. Numbers in the shaded area indicate the percentage of isolates that had an MIC greater than the highest concentration tested. * Amoxicillin shown, clavulanic acid at 1/2X concentration that of amoxicillin. ** Trimethoprim shown, sulfamethoxazole at 19X concentration that of trimethoprim. ^1^ Percent of the isolates that were resistant out of the total 1,050 non-type-specific *E. coli* isolates tested. ^2^ 95% confidence interval was calculated using exact binomial method. ^▵^ One-sided 97.5% confidence interval; used only when estimate was zero.

Two to three distinct NTS *E. coli* colonies were chosen from the TSA plates and suspended into 4 ml of sterile deionized water to adjust to a 0.5 McFarland standard. Ten microliters of the suspension was mixed with Mueller-Hinton broth, and 50 µl of the suspension was inoculated to each well of a Sensititre plate using the Sensititre™ automated inoculator (Trek Diagnostic Systems, Cleveland, OH). The plates were incubated at 37°C for 18 hours. Minimum inhibitory concentrations (MIC) of 15 different antibiotics were determined *via* the broth micro-dilution method using the Gram-negative National Antimicrobial Resistance Monitoring System (NARMS) panel CMV1AGNF (Trek Diagnostic Systems, Cleveland, OH) [28]. The plates were read by the Sensititre ARIS® automated system (Trek Diagnostic Systems, Cleveland, OH). The Sensititre ARIS® automated system interprets isolates as susceptible, intermediate, or resistant according to Clinical and Laboratory Standards Institute (CLSI) guidelines [28]. For our purposes, isolates demonstrating intermediate susceptibility toward antimicrobials were re-classified as susceptible to allow a binary classification in data analysis. For those antibiotics without breakpoints established by the CLSI guidelines, we instead used ‘consensus’ breakpoints established by the NARMS for enteric bacteria [29] (see Figure 2). *Escherichia coli* ATCC 25922, *Escherichia coli* ATCC 35218, *Pseudomonas aeroginosa* ATCC 27853, *Staphylococcus aureus* ATCC 29213, and *Enterococcus faecalis* ATCC 29212 (American Type Culture Collection, Manassas, VA) were used as quality control strains for susceptibility testing. The MIC results obtained from these quality control strains were compared with the quality control ranges recommended by the CLSI [28]. Quality control was performed for every new batch of Mueller-Hinton broth used and for every new batch of microbroth dilution susceptibility plates.

### Detection of resistance genes

A duplex PCR assay to detect both *tet*(A) and *tet*(B) was performed as previously described [30] using DNA extracted from the same 1,050 NTS *E. coli* isolates for which the antibiotic susceptibility test was conducted. The *bla*_CMY-2_ PCR was performed as previously described [31]. The primers used for all PCR reactions are listed in Table 2. Promega® PCR mastermix (Promega Corp., Madison, WI) was used for both assays. All reactions were carried out in Eppendorf Mastercycler® gradient thermal cyclers (USA Scientific, Inc., Ocala, FL). Automated capillary electrophoresis analysis of the PCR product for all three resistance genes was performed *via* the QIAxcel System (QIAgen, Valencia, CA).

**Table 2:** PCR primers used for PCR reactions.

Gene name	Primer	Primer Sequence	Expected Product Size (bp)	GenBank Accession no.*
*bla*_CMY-2_	585F	5′- CAG ACG CGT CCT GCA ACC ATT AAA -3′	454 a	AB212086
	1038R	5′- TAC GTA GCT GCC AAA TCC ACC AGT -3′		
*tet*(A)	*tet*(A)(F)	5′ -GCTACATCCTGCTTGCCTTC- 3′	210 b	X61367
	*tet*(A)(R)	5′ -CATAGATCGCCGTGAAGAGG- 3′		
*tet*(B)	*tet*(B) (F)	5′ -TTGGTTAGGGGCAAGTTTTG- 3′	659 b	J01830
	*tet*(B) (R)	5′ -GTAATGGGCCAATAACACCG- 3′		

a Primer set used is from [31].

b Primer set used is from [54].

* Sequence used for primer design.

Controls: The positive control used for the duplex reaction was a 1∶1 mixture of the DNA obtained from *E. coli* ATCC 47042 and the XL1-Blue *E. coli* strain. *E. coli* ATCC 47042 is known to harbor the *tet*(B) gene, and the XL1-Blue *E. coli* strain harbors the *tet*(A) gene. *E. coli* strain (M1) was used as the positive control for the *bla*_CMY-2_ gene. It was previously obtained from the University of Illinois, Chicago [32]. This strain is known to harbor the *bla*_CMY-2_ gene. The negative control consisted of the mastermix alone.

### Statistical Methods

#### Descriptive Statistics

The outcome measures (and data types) were: 1) proportion of resistant (versus susceptible) NTS *E. coli* isolates for each of 15 antimicrobials (binary), 2) log_2_ MIC for each of 15 antimicrobials (truncated integer), and 3) presence (or absence) of three different resistance genes (binary). Basic descriptive statistics were computed by cross-tabulating each of these outcomes across four sampling days for each treatment group. The phenotypic MDR count of an isolate was determined by establishing the total number of antimicrobials, out of the 15 antimicrobials tested on the NARMS panel, to which an isolate was phenotypically resistant. The overall frequency distribution of the phenotypic MDR counts among isolates in all four groups was examined. These distributions were compared to evaluate the treatment effect. Similarly, the presence (or, absence) of the three resistance genes was cross-tabulated by treatment and day, as well as with the phenotypic MDR counts obtained from the NARMS panel. Significance of associations was determined by likelihood ratio chi-square test. Distributions of MIC for each of the 15 antimicrobials, cross-tabulated by treatment and day, were also examined.

#### Multivariable analyses

Generalized estimating equations (GEE) with binomial error distribution and logit link functions were used to analyze the data (STATA® SE Release 12.1; STATA Corp., College Station, TX). This approach was used to simultaneously evaluate the risk factors for isolates exhibiting phenotypic expression of ceftiofur and tetracycline resistances, and for isolates harboring *bla*_CMY-2_, *tet*(A), and *tet*(B) genes separately. All GEE models were adjusted for the pen-level dependencies assuming exchangeable correlation structures at the pen level. Dependencies were also expected among the three isolates derived from a single fecal sample on each day; however, pen- and animal-level dependencies both could not be accounted for simultaneously due to convergence problems in a multi-level mixed logistic model (XTMELOGIT in STATA®) that was attempted before settling on a more robust GEE framework.

The factors that resulted in isolates with higher phenotypic MDR counts (number of antimicrobials toward which an isolate exhibited resistance) were analyzed using ordinal logistic models. Resistances were exhibited by isolates to a maximum of 12 out of 15 antimicrobials in this study. Therefore, there were 13 different categories (e.g., pan-susceptible, single-, double-, penta-, deca-, dodeca-resistant) depending on the number of antimicrobials to which an isolate exhibited resistance.

Logistic regression models for discrete-time survival analysis were used to model treatment factor effects on the ability of NTS *E. coli* isolates to grow/survive over each of the increasing ceftiofur concentrations, as tested on the NARMS panel [33]. This approach allowed for right-censoring of the MIC data at the highest recorded concentration present on the panel.

## Results

### Descriptive statistics

The distributions of MICs for all 15 antibiotics among the 1,050 NTS *E. coli* isolates tested are shown in Figure 2. Out of 1,050 NTS *E. coli* isolates, resistance to at least one antimicrobial was detected in 710 (67.62%) isolates, and 340 (32.38%) isolates were found to be susceptible to all 15 antimicrobials tested. The most common resistance was detected against tetracycline (642 [61.14%] isolates), ampicillin (391 [37.24%] isolates), sulfisoxazole (329 [31.33%] isolates), streptomycin (308 [29.33%] isolates), ceftriaxone (267 [25.43%] isolates), chloramphenicol (246 [23.43%] isolates), and ceftiofur (241 [22.95%] isolates), as shown in Figure 2.

**Figure 3:**
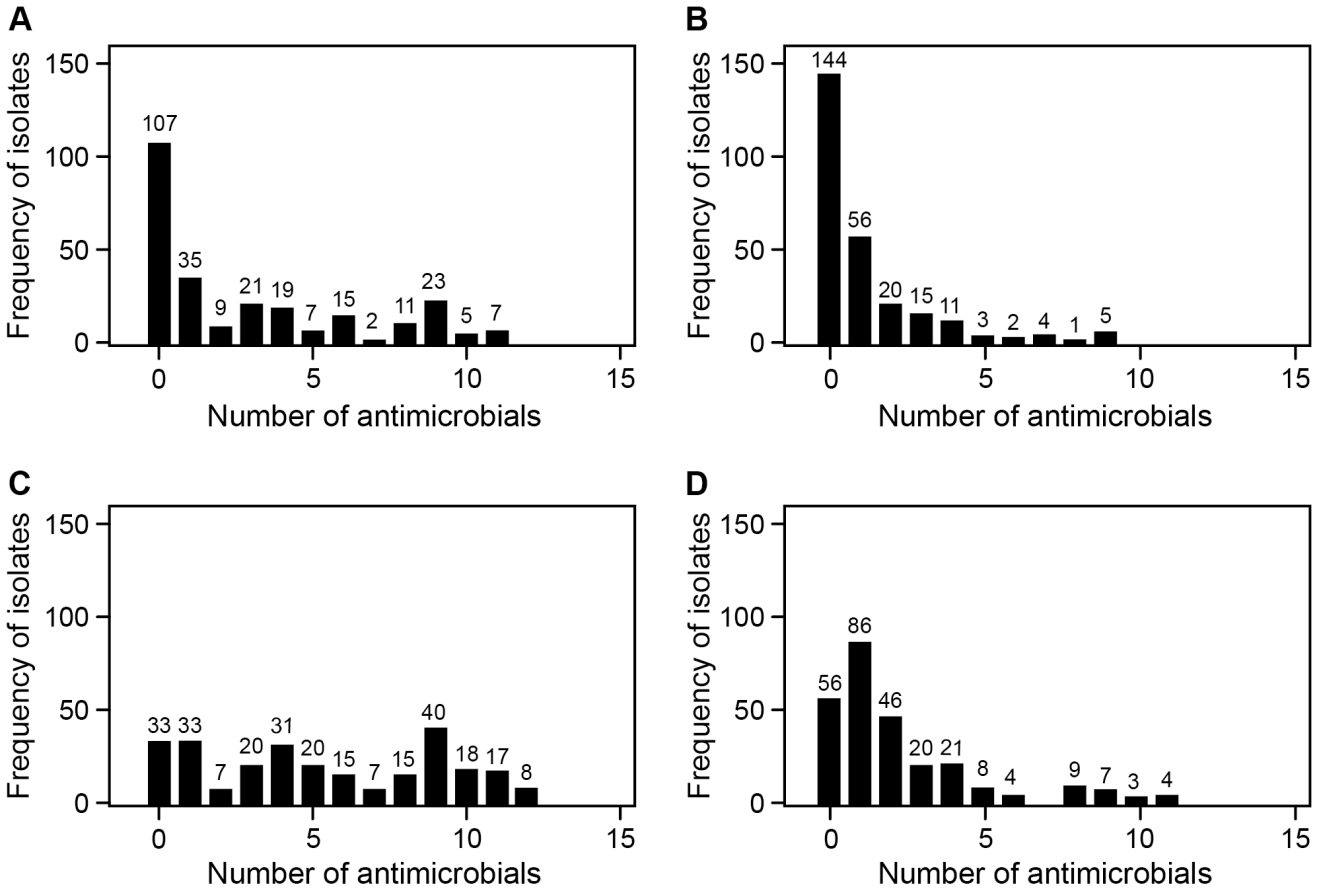
Frequency distribution of *E. coli* by phenotypic multidrug resistance counts for the four treatment groups. (A) CCFA administered to all steers within pens without subsequent CTC administration at the pen level; (B) CCFA administered to one out of 11 steers within pens without subsequent CTC administration at the pen level; (C) CCFA administered to all steers within pens followed by CTC administered at the pen level; (D) CCFA administered to one out of 11 steers within pens followed by CTC administered at the pen level.

The frequency distribution of the *E. coli* isolates by the phenotypic MDR count for all four treatment groups is shown in Figure 3. Pens in which CCFA was administered to 1 out of 11 steers and CTC treatment was not administered were exposed to the least antimicrobial selection pressure (Figure 3B). Within this treatment group, 144 (55.1%) of the isolates were pan-susceptible to the panel of 15 antimicrobials; in addition, the distribution was highly right-skewed, indicating decreasing numbers of greater phenotypic MDR counts in this group (Figure 3B). A higher prevalence of greater phenotypic MDR counts was identified in isolates from animals within pens where CCFA was administered to all the animals but that were not subsequently fed CTC in feed (Figure 3A and B). The frequency distribution of phenotypic MDR counts in this treatment group (illustrating CCFA treatment alone) was more uniformly distributed over the full range of MDR numbers (0–15); in this case, 41% of the isolates were found to be pan-susceptible to all 15 antimicrobials (Figure 3A). The effect of CTC alone was best illustrated by the contrasts among the pens, in which only 1 among 11 animals had prior CCFA exposure (Figure 3B and D). Chlortetracycline exposure increased the prevalence of higher phenotypic MDR counts (Figure 3D); however, CTC tended to select for lower MDR categories (isolates resistant to one or two antimicrobials) rather than categories with ≥5 antimicrobials. The CTC effect was much more profound when it followed CCFA treatment applied to all animals in a pen (Figure 3C). These pens illustrated the maximum CCFA and CTC treatment effects (Figure 3C), and their isolate profiles illustrated a highly uniform distribution with a quite remarkable 3.03% of NTS *E. coli* isolates resistant to 12 antimicrobials.

**Figure 4:**
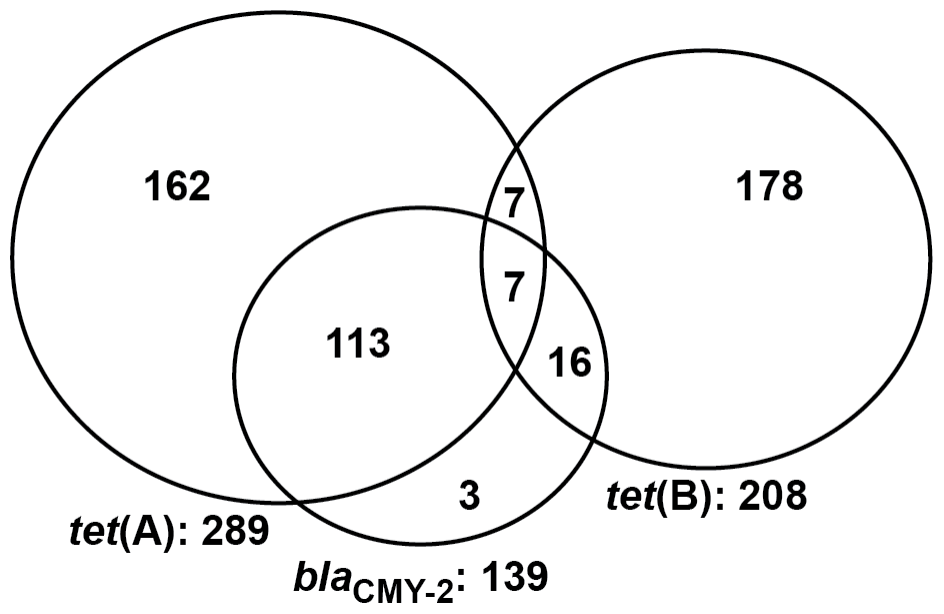
Proportional Venn diagram illustrating the joint frequencies of three resistance genes among *E. coli* isolates. Bolded numbers represent the marginal totals for each of the *tet*(A), *tet*(B), and *bla*_CMY-2_ genes among 1,050 NTS *E. coli* isolates. A total of 564 isolates did not harbor any of the three genes.

Overall 289, 208, and 139 NTS *E. coli* isolates harbored *tet*(A), *tet*(B), and *bla*_CMY-2_ genes, respectively (Figure 4). These three genes were not detected in the remaining 564 isolates. Only three isolates harbored *bla*_CMY-2_ alone; that is, without *tet*(A) or *tet*(B). The majority of *bla*_CMY-2_ positive isolates also harbored the *tet*(A) gene (n = 120), whereas the *bla*_CMY-2_ gene was much less prevalent among the isolates also harboring the *tet*(B) gene (n = 23). Only 14 isolates harbored *tet*(A) and *tet*(B) genes together; further, there were seven isolates which harbored all the three resistance genes (Figure 4).

**Figure 5:**
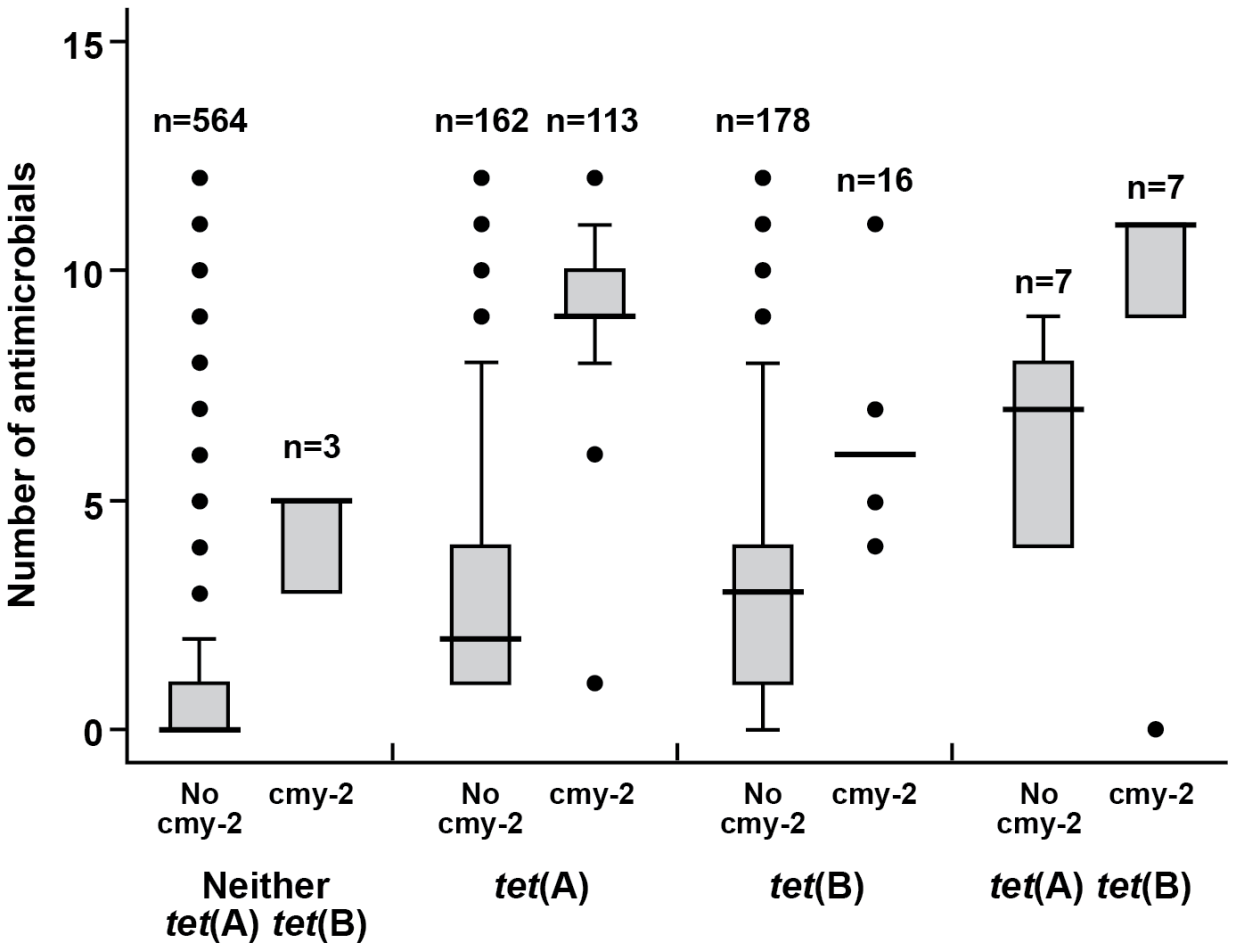
Box plot of the phenotypic multidrug resistance count cross-tabulated by different resistance gene combinations. Legend terms: cmy-2, no cmy-2, *tet*(A), *tet*(B), *tet*(A)*tet*(B), neither *tet*(A) *tet*(B) in the figure are used for isolates that were found to be positive for *bla*_CMY-2_, negative for *bla*_CMY-2_, positive for *tet*(A), positive for *tet(*B), positive for both *tet*(A) and *tet*(B), and negative for *tet*(A) and *tet*(B) genes, respectively. Horizontal bars indicate the median number of antimicrobials among each of gene combinations.

The association of a particular gene or gene combination with the phenotypic MDR count was evaluated and illustrated by way of the box plot (Figure 5). Overall, the presence of the *tet*(A) gene (n = 289) was associated with isolate phenotypes exhibiting reduced susceptibility to a higher median number of antimicrobials (median = 6, 95% CI = 4–8) compared with the *tet*(B) gene (n = 208; median = 3, 95% CI = 3–4). Similarly, *bla*_CMY-2_ gene-positive isolates were associated with very high phenotypic MDR count (n = 139; median = 9). The 564 isolates that were found to be negative for all three resistance genes were generally pan-susceptible (median number of antimicrobials = 0) (Figure 5). The 162 isolates harboring only *tet*(A), in the absence of *tet*(B) and *bla*_CMY-2_, exhibited phenotypic resistance toward a median number of two antimicrobials. Isolates, when positive for both *tet*(A) and *bla*_CMY-2_ and in the absence of *tet*(B) (n = 113), exhibited a higher phenotypic MDR count (median  = 9 antimicrobials); on the other hand, isolates positive for only *tet*(B), but in the absence of *tet*(A) and *bla*_CMY-2_ (n = 178), were resistant to a median of three antimicrobials. In Figure 5, the box plot graphic clearly demonstrates that the presence of the *bla*_CMY-2_ gene was associated with higher phenotypic MDR counts.

### Multivariable analyses

Generalized estimating equation (GEE) with three-way full factorial designs were used to evaluate the effects of the treatment strategies on resistance, measured both at genotypic and phenotypic levels. The three factors corresponded to CCFA administered to either one or all the animals within pens (Mix; binary variable), CTC administration to all animals within a pen (CTC; binary variable), and study period (Day 0, 4, 12, or 26; categories coded as an indicator variable with 0 as referent), respectively. Results were interpreted at the pen-level, not at the individual-animal level.

**Figure 6:**
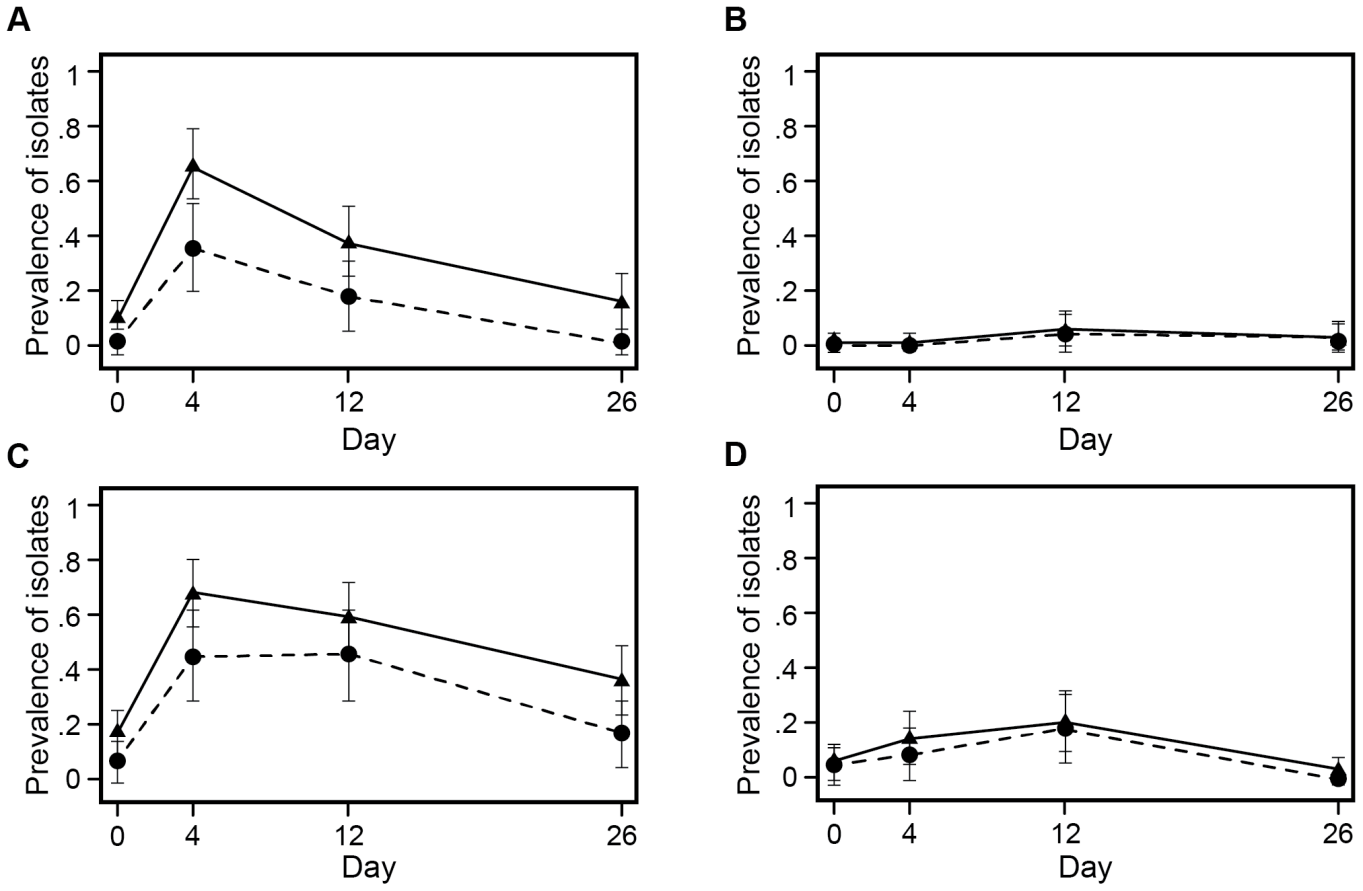
Prevalence of ceftiofur-resistant *E. coli* isolates, modeled as marginal predicted probabilities, over days. Solid line represents the proportion of NTS *E. coli* isolates phenotypically resistant to ceftiofur at ≥8 µg/ml. Dashed line represents the proportion of isolates harboring the *bla*_CMY-2_ gene. The four treatment groups are (A) CCFA administered to all steers within pens without subsequent CTC administration at pen level; (B) CCFA administered to 1 out of 11 steers within pens without subsequent CTC administration at pen level; (C) CCFA administered to all steers within pens followed by CTC administered at pen level; (D) CCFA administered to 1 out of 11 steers within pens followed by CTC administered at pen level.

Figure 6 illustrates the prevalence of NTS *E. coli* isolates exhibiting both phenotypic resistance to ceftiofur (shown by the solid line) and the presence of *bla*_CMY-2_ (shown by the dashed line). The likelihood of recovering ceftiofur-resistant NTS *E. coli* or *bla*_CMY-2_ positive isolates tended to increase following CCFA administration (Figure 6 A, C; Day 4). Chlortetracycline treatment delayed the return of ceftiofur resistance to the baseline (Figure 6 C, D; Day12). In fact, CTC appeared to favor expansion of the ceftiofur-resistant population, fully independent of the prior CCFA regimen. Importantly, phenotypic ceftiofur resistance was not completely associated with the presence of *bla*_CMY-2_ gene at all time points, nor in all four treatment groups. Multiple isolates (n = 102) exhibited phenotypic ceftiofur resistance but did not harbor the *bla*_CMY-2_ gene. This can best be appreciated by the gap between the line graphs illustrating phenotypic versus genotypic (*bla*_CMY-2_) ceftiofur resistance in Figure 6. Further characterization of the non-*bla*_CMY-2_ isolates has been carried out to explain this difference, and results were reported elsewhere [34].

**Figure 7:**
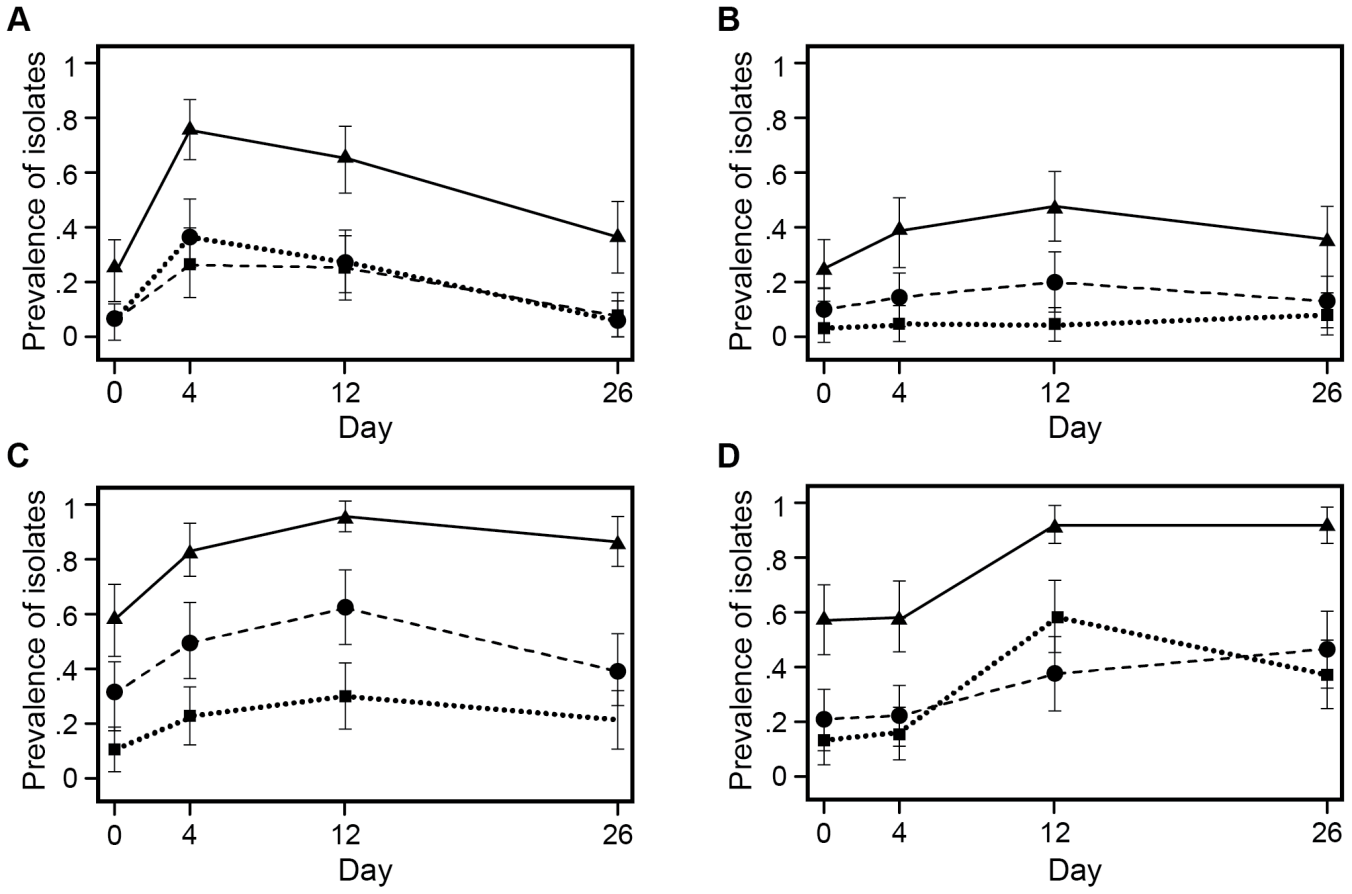
Prevalence of tetracycline-resistant *E. coli* isolates, modeled as marginal predicted probabilities, over days. Solid line represents the proportion of NTS *E. coli* isolates expressing phenotypic tetracycline resistance; dashed line represents the proportion of NTS *E. coli* isolates harboring the *tet*(A) gene; dotted line represents the proportion of NTS *E. coli* isolates harboring the *tet*(B) gene. The four treatment groups are: (A) CCFA administered to all steers within pens without subsequent CTC administration at pen level; (B) CCFA administered to 1 out of 11 steers within pens without subsequent CTC administration at pen level; (C) CCFA administered to all steers within pens followed by CTC administered at pen level; (D) CCFA administered to 1 out of 11 steers within pens followed by CTC administered at pen level.

The likelihood of recovering isolates expressing tetracycline resistance, as well as isolates harboring tetracycline resistance genes, increased with CCFA administration (Figure 7 A, C; Day 4). As expected, CTC treatment further increased tetracycline resistance, both at phenotypic and genotypic levels (Figure 7 C, D; Day 12). Importantly, there was a differential selection favoring isolates harboring *tet*(A) over *tet*(B) following CCFA treatment administered to all steers within a pen (Figure 7 C; Day 4). However, when only 1 steer in a pen of 11 animals received prior CCFA treatment, there was a clear preferential selection favoring isolates harboring *tet*(B) gene over *tet*(A) once CTC was administered in the feed (Figure 7 D; Day 12).

**Figure 8:**
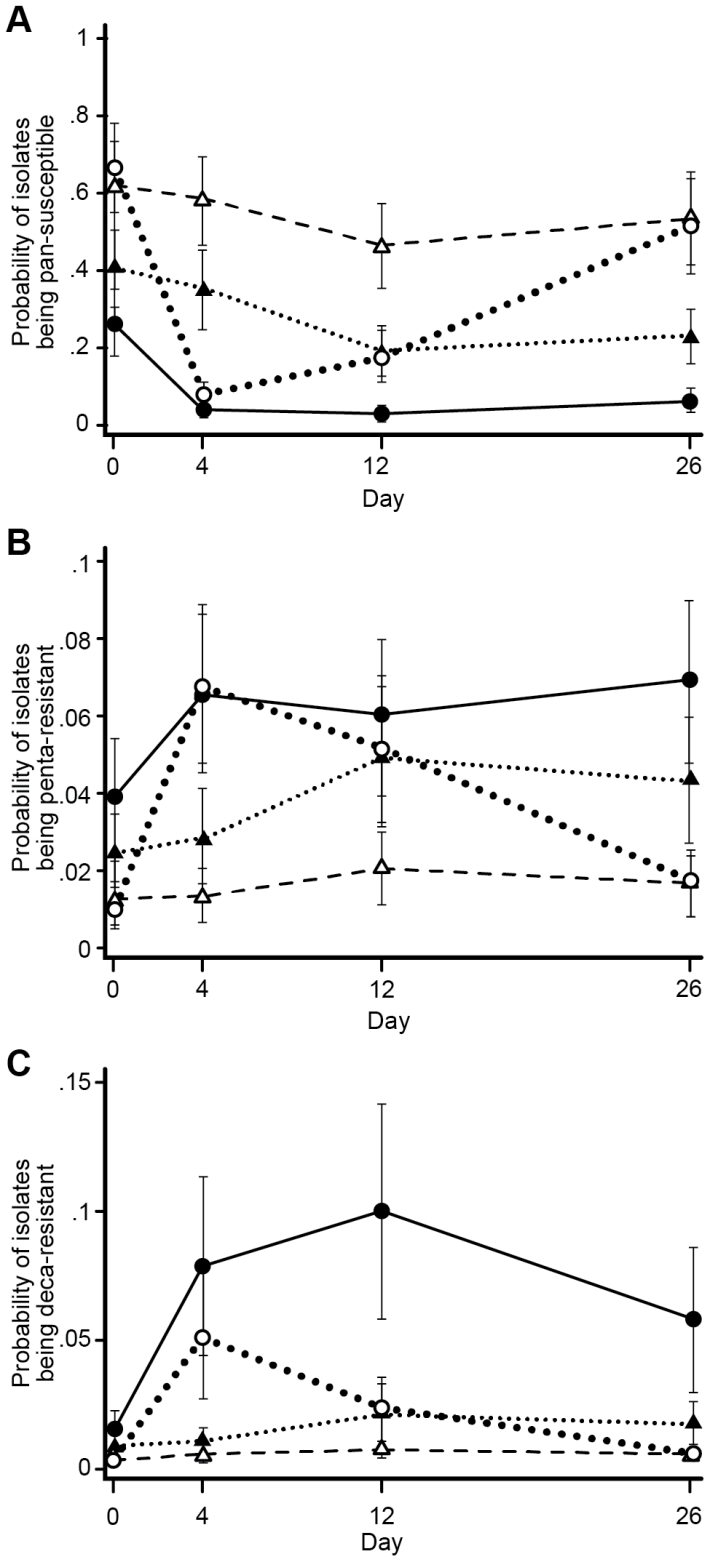
Probabilities of *E. coli* isolates to be pan-susceptible, penta-resistant, or deca-resistant among treatment groups. The three sub-graphs represent modeled marginal predicted probabilities for *E. coli* isolates to be (A) pan-susceptible, (B) penta-resistant, or (C) deca-resistant among the four treatment groups. The four treatment groups are represented by four lines in all three sub graphs. Dotted (large) line represent pens with CCFA administered to all steers within pens without subsequent CTC administration at pen level; Dashed line represents CCFA administered to 1 out of 11 steers within pens without subsequent CTC administration at pen level; solid line represents CCFA administered to all steers within pens followed by CTC administered at pen level; dotted (small) line represents CCFA administered to 1 out of 11 steers within pens followed by CTC administered at pen level.

The association between treatment strategies and the phenotypic MDR count was assessed using an ordinal logistic model with a three-way full factorial design (Mix, CTC, and Day) as outlined above. Phenotypic MDR count was defined in this study as the number of antimicrobials, out of the panel of 15 antimicrobials tested on the NARMS panel, toward which an isolate exhibited (binary) resistance. The treatment groups in which all steers received CCFA were observed to have a significant decrease in the proportion of isolates that were pan-susceptible (Figure 8 A, Day 4). There also was a significant effect of CCFA treatment on increasing the likelihood of recovering penta- or deca-resistant isolates (Figure 8 B & C, Day 4). This was in marked contrast to the groups in which only 1 animal among 11 in a pen received CCFA treatment. Less markedly, but also significant, was that feeding of CTC lowered the probability of isolates being pan-susceptible compared with pens of cattle that did not receive CTC (Figure 8 A, Day 12). Chlortetracycline administration was also associated with increased odds for recovering both penta- and deca-resistant isolates, and this was especially notable when prior CCFA treatment was administered to only 1out of 11 steers within the pen (Figure 8 B & C, Day 12). When CCFA was administered to all steers, subsequent CTC administration appeared to sustain the penta-resistant proportion while further increasing the deca-resistant NTS *E. coli* proportion of isolates. Overall, CCFA appeared to have the more dramatic effect on selection of higher phenotypic MDR counts (penta- or deca- resistant) than CTC alone; however, CTC greatly exacerbated the prior effects of CCFA on phenotypic MDR counts.

**Figure 9:**
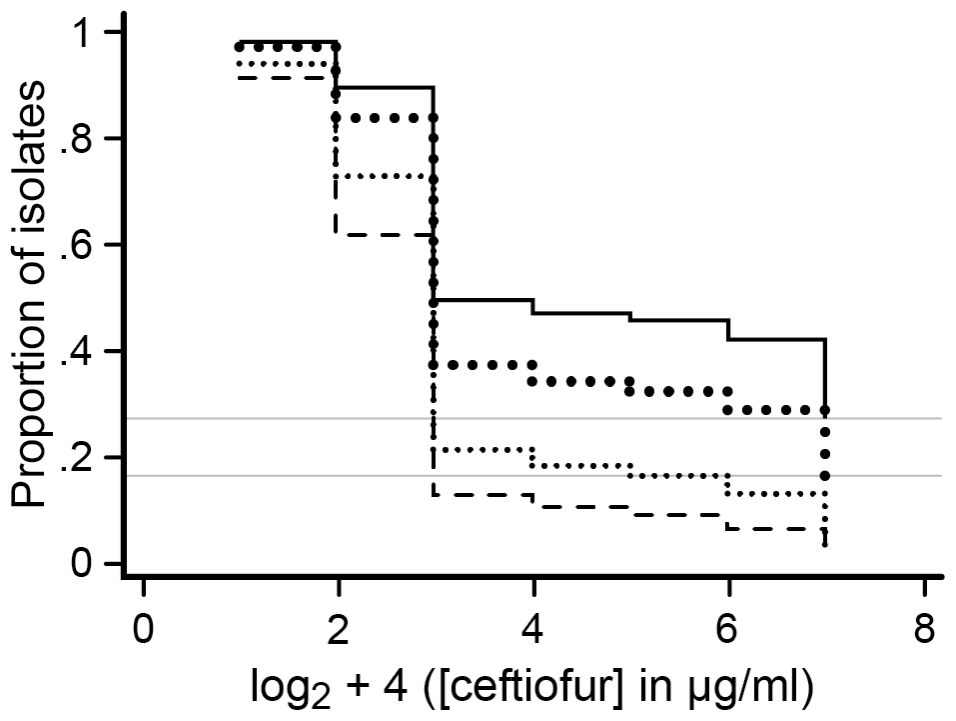
Survival curve of *E. coli* isolates over each increasing ceftiofur concentrations tested, among treatment groups. Each survival curve represents the predicted probabilities of growth of NTS *E. coli* isolates on each of the increasing ceftiofur concentration tested on the NARMS plate as shown on a log scale in the figure. Dotted (large) line represent pens with CCFA administered to all steers within pens without subsequent CTC administration at pen level; dashed line represents CCFA administered to 1 out of 11 steers within pens without subsequent CTC administration at pen level; solid line represents CCFA administered to all steers within pens followed by CTC administered at pen level; dotted (small) line represents CCFA administered to 1 out of 11 steers within pens followed by CTC administered at pen level.

A discrete-time logistic model was used to compare the proportion of NTS *E. coli* isolates that were able to grow (i.e., were not inhibited) over each of the increasing ceftiofur concentrations on the NARMS panel among the four treatment groups. The fixed effects for the model consisted of Mix, CTC, and ‘survival time’ (survival time here was an analog for each increasing concentration of ceftiofur tested on the NARMS panel). The two-way interactions with ‘time’ were statistically insignificant in the model and therefore were excluded from the final model. Survival curves represent the predicted probabilities of NTS *E. coli* isolates not being inhibited at each increasing concentration of ceftiofur; ceftiofur concentration is graphed as zero-adjusted and ordered log_2_ transformed (MIC) values for all four groups (Figure 9). The two reference lines in Figure 9 indicate that the two treatment groups in which all steers received CCFA treatment had 26% and 17% of isolates that were not inhibited even at the highest concentrations of the ceftiofur tested on the NARMS plate (log_2_ (8 µg/ml) +4 = 7). These proportions were considered right-censored and were significantly higher than in the groups in which only 1 animal among 11 in a pen received ceftiofur treatment. The steers in CTC-administered pens had a similarly higher proportion of isolates that were not inhibited at higher concentrations of ceftiofur compared with the isolates derived from pens in which CTC was not administered.

## Discussion

This study was designed to evaluate the effects of co-housing ceftiofur treated and non-treated animals and the effect of CTC treatment following ceftiofur treatment. The effects were measured in terms of the phenotypic susceptibility profiles of NTS *E. coli* isolates and by evaluating the genotypic prevalence of specific resistance genes among these same NTS *E. coli* isolates.

CCFA treatment on Day 0 selected for isolates with reduced susceptibility towards ceftiofur, both at genotypic and phenotypic levels. Four other major published studies have evaluated the effect of ceftiofur on cephalosporin resistance among enteric bacteria in cattle [20], [35], [36], [37]. Our results were quite similar to two of these earlier studies [20], [36], which indicated that CCFA was associated with a significant decrease in the total *E. coli* log_10_ CFU/g of feces in cattle. This combined with a concurrent, although transient, expansion of the ceftiofur-resistant *E. coli* fraction following CCFA administration, resulted in an observed increase in prevalence [20], [36]. In our study, the total *E. coli* CFU/g of feces also dropped by approximately two logs following CCFA treatment (data not shown) and remained lower than baseline for approximately 8–12 days [38]. A third study [35] reported a similar transient increase in the animal-level prevalence of ceftiofur resistant *E. coli* immediately following ceftiofur treatment. However, they did not observe a herd-level association between levels of ceftiofur use and increased shedding of extended-spectrum cephalosporin-resistant *E. coli* isolates. These authors did not report the variations in the total *E. coli* load among samples collected during the study period; thus, their results could have been biased by ignoring the decrease in background susceptible bacterial population. The fourth study [37] did not observe the animal-level associations between the ceftiofur use and prevalence of *E. coli* isolates with reduced susceptibility to ceftriaxone; however, while a significant herd-level association was associated with use versus non-use, no dose-response was detected among herds that used ceftiofur. This was a cross-sectional prevalence study conducted in only 18 dairy herds. Herds reporting ceftiofur use had a significantly higher prevalence of ceftriaxone resistance isolates among cattle fecal samples than the herds that did not use ceftiofur. The individual ceftiofur-treated cattle themselves did not exhibit an increased risk of shedding of *E. coli* isolates that had reduced susceptibility to ceftriaxone. This result may easily be attributed to the study design; the time duration between ceftiofur treatment and fecal sample collection was not controlled. The increase in shedding of resistant isolates is typically found to be transitory, and resistance levels usually return to baseline levels soon after selection pressures are removed [20], [36]. Therefore, the probability of finding a significant relationship between ceftiofur treatment and resistant bacteria in the post-antimicrobial washout phase is low within a pen or herd. On the other hand, herd-level associations suggest that a significant treatment- and time-dependent shift in ceftriaxone resistance baseline levels could occur after a product is first introduced into a herd [20].

In contrast to our hypothesis, subsequent CTC treatment starting on Day 4 was not associated with a decrease in ceftiofur resistance as was seen in a previous study [19]. Instead, CTC greatly delayed the return of ceftiofur resistance to baseline levels following CCFA treatment. Steers from both studies were housed in the same research feedlot at West Texas A&M University. The bacterial load was not quantified in the previous study. In the present study, the total NTS *E. coli* load was quantified by measuring CFU counts [38]. It was determined that, overall, CTC treatment slightly increased the NTS *E. coli* population. Therefore, CTC treatment might also have led to an expansion of the ceftiofur-resistant population instead. This was in direct contrast to the hypothesis of the present study.

Although the results were unexpected, they can be explained by simple mechanistic considerations of co-selection. The genotypic analysis of the isolates obtained from this study revealed certain associations: CTC treatment in the absence of prior CCFA treatment significantly increased selection of the *tet*(B) gene over the *tet*(A) gene; the two *tet* genes were found to be negatively associated with each other; and the *tet*(A) gene was found to be strongly associated with the *bla*_CMY-2_ gene. The CTC treatment in the previous study [19] may have differentially selected for isolates with *tet*(B) genes over *tet*(A) gene. The *tet*(B) and *bla*_CMY-2_ genes, not being significantly associated with each other, may have led to the finding of the previous study suggesting that CTC treatment resulted in a preferential selection of tetracycline resistant isolates over isolates co-resistant to both tetracycline and ceftiofur [19]. However, in the present study, pens (or animals) receiving CCFA treatment selected for *bla*_CMY-2_ positive isolates. This expanded population likely also favored the *tet*(A) over the *tet*(B) gene. Subsequent CTC treatment in the present trial may have further expanded this population (isolates harboring *tet*(A) along with *bla*_CMY-2_ genes); therefore, the reduction in ceftiofur resistance was not seen in these pens. A major difference between the present study and that of Platt *et al.* (2008) was that the latter did not include prior ‘priming’ of the bacterial population with CCFA, either at the pen-level or of single individuals within those pens. Furthermore, the ‘baseline’ prevalence of resistance to ceftiofur in the experimental feedlot has increased steadily over the years from 2003 through the present as traced from Lowrance *et al.* (2007) [20] through Platt *et al.* (2008) [19]. Although one of our treatment groups closely mimicked the previous study [19]; that is, where CCFA was administered to 1 among the 11 steers within pens and then later on Day 4 all animals were exposed to CTC treatment, CTC in those pens too was not observed to reduce ceftiofur resistance. The present study was designed to evaluate the effects of CTC as an intervention strategy to control ceftiofur resistance, and the difference in study design compared with Platt *et al.* may have caused some of the disparity. However, it is extremely unlikely to have caused all of the disparity. The *E. coli* isolates from both studies are being further characterized to better explain the study discrepancies, especially as relates to the earlier assumptions about the expected associations among *tet*(A), *tet*(B), and *bla*_CMY-2_ genes.

Over the entire study period, the frequency of phenotypically ceftiofur-resistant NTS *E. coli* isolates was always higher than the prevalence of isolates harboring *bla*_CMY-2_. All earlier work in North America suggested that the *bla*_CMY-2_ gene dominated and explained almost 100% of the resistance to ceftiofur in feeder cattle populations [24], [25]. As time moves forward, it is expected that there can be other genetic determinants such as ESBL genes that will contribute to explain the disparity between phenotypic expression and genotype. One previous study [39] indicated that the phenotypic and genotypic disparity could be attributed to other resistance determinants that were not tested for or could be due to the inability of the resistance genes to be turned on, in order to express phenotypic resistance. The isolates in the present trial were further characterized to explain the observed discrepancy between phenotypic expression and genotype. Twenty-nine out of 88 steers tested positive for the *bla*_CTX-M-32_ gene over the study period [34]. The presence of the *bla*_CTX-M-32_ gene and *ampC* promoter mutation among NTS *E. coli* isolates almost completely explains the higher frequency of phenotypic ceftiofur resistance observed among isolates as opposed to that predicted by *bla*_CMY-2_ alone.

CCFA treatment on Day 0 also selected for isolates with reduced susceptibility towards tetracycline. A similar effect has been observed elsewhere [20], [40]. Also at the genotypic level, the prevalence of isolates harboring tetracycline resistance genes increased with CCFA treatment. Chlortetracycline treatment further increased the frequency of tetracycline resistance. In this study, in the situation where the vast majority of steers in a pen were not primed with CCFA before CTC treatment, there was a differential selection of isolates harboring the *tet*(B) gene over the *tet*(A) gene (Figure 7 D). Chlortetracycline treatment effects on tetracycline resistance prevalence have been previously investigated [19], [41]. One of those studies [41] detected no significant increase in tetracycline resistance following CTC treatment. The authors attributed this result to the gap in sampling time and a low initial prevalence of tetracycline resistance. The other study [19] observed a significant increase in the likelihood of recovering tetracycline resistant isolates during and immediately following CTC administration. The effect was transitory, and the prevalence of the isolates with reduced susceptibility returned to baseline levels by 17 days post-treatment.

Pens in which all animals received CCFA treatment had significantly higher pen-level ceftiofur resistance compared with pens in which only 1 of 11 animals were CCFA-treated. A previous study [42] in which ceftiofur-treated and non-treated dairy cattle were co-housed, reported a small increase in the *bla*_CMY-2_ gene copy numbers in fecal community DNA of the non-treated animals. The authors attributed this effect to co-mingling of treated and non-treated animals. Those results suggested horizontal transmission of bacterial strains/resistant determinants among the cattle that were housed together. In our study, the non-treated animals were expected to supply susceptible enteric bacteria into the environment within pens when treated and non-treated animals were housed together. The treated animals also were in constant exposure to these susceptible bacteria. These bacteria were expected to improve the microbial ecology of the treated animals by more rapidly returning the gut flora to baseline or equilibrium levels of resistance. However, the present study was not designed to evaluate the animal-level effects of co-housing of treated and non-treated animals; rather, the effects were assessed solely at the pen-level with sufficient statistical power to meet our stated objectives. Further studies are required to establish any individual animal-level effects because insufficient statistical power is present in this study design to evaluate ‘mixing’ effects on the individual steers (n = 4 total) receiving CCFA among 11 in a pen.

Antimicrobial pressure exerted both by CCFA and CTC selected for isolates with higher phenotypic MDR counts. The effects of CCFA on co-selecting for other phenotypic antimicrobial resistances besides cephalosporins were more profound than CTC treatment. At the genotypic level, the presence of the *bla*_CMY-2_ gene seemed to have a similar effect on co-selection. Isolates harboring the *bla*_CMY-2_ gene showed phenotypic resistance to a higher median number of antimicrobials when directly compared with isolates that did not harbor this gene. Studies in the United States on *Salmonella* isolates derived from bovine, porcine, and human origin have indicated that the *bla*_CMY-2_ gene is usually located on a large IncA/C plasmid that harbors several other resistance genes [21], [22], [43]. Overall across all treatment groups and days, isolates harboring the *tet*(A) gene also showed phenotypic resistance against a higher median number of antimicrobials than those isolates carrying the *tet*(B) gene. One previous study instead indicated an association of *tet*(B) genes with more multiple drug resistances when compared with isolates harboring the *tet*(A) gene [44]. However, that was a cross-sectional prevalence study conducted at the farm-level. In this randomized controlled trial, pretreatment (Day 0) results suggested no significant difference between these two *tet* genes with respect to the carriage of multiple phenotypic antimicrobial resistance. CCFA administered on Day 0 selected for isolates harboring *bla*_CMY-2_, and this gene was associated with resistance against many other antimicrobials. Importantly, the *bla*_CMY-2_ gene exhibited a positive association with the *tet*(A) gene. There was likely to have been co-selection of isolates harboring *bla*_CMY-2_ and *tet*(A) genes because of the initial CCFA treatment. It is also possible that isolates harboring the *tet*(A) gene demonstrated a higher multidrug phenotypic resistance count due to prior CCFA exposure and selection. Therefore, in our study *tet*(A) positive isolates exhibited an overall higher MDR profile than *tet*(B) positive isolates.

A negative association was found between *tet*(A) and *tet*(B) genes among NTS *E. coli* isolates. Only 0.01% (14 isolates) harbored both the *tet*(A) and *tet*(B) gene. Previous studies also have indicated a negative association between these two *tet* genes [39], [44], [45]. The negative association between the tetracycline resistance determinants has been suggested by some to be due to the incompatibility of the plasmids that carry these genes [46]. This possibility is currently being investigated *via* plasmid-typing of these 1,050 isolates. Only three isolates harbored only the *bla*_CMY-2_ gene without the presence of either the *tet*(A) or *tet*(B) gene. Earlier studies in North American cattle have reported that the *bla*_CMY-2_ gene is usually present with at least one other resistance gene. However, we must acknowledge the high probability that there were other unexamined resistance factors present with the gene (beyond those few we examined); in fact, the phenotypic resistance profile suggests this was likely to have been the case, although not always to have been associated with the usual IncA/C plasmid [34], [47].

The present system of classifying antimicrobial susceptibility data into susceptible or resistant has been critiqued before [41], [48]. Such a system does not easily allow for analyzing the trends of changes in MIC values statistically. The changes in the MIC values, both above and below an internationally accepted cut point (e.g., CLSI in North America; EUCAST in Europe), cannot readily be evaluated by the binary coding system. In addition, such cut points are subject to change, especially when they are not based on epidemiological breakpoints. Survival analyses using non-parametric assumptions such as the Kaplan-Meier method [49], [50] or Cox proportional hazards model [48] have been proposed as alternative approaches for analyzing MIC data. These methods use the entire dilution range of antimicrobials tested and also deal effectively with the large number of right-censored observations (i.e., isolates that grow beyond the upper limit of antibiotic concentration included on commercially available plates). The resulting survival curves illustrate and compare the proportion of bacteria that are uninhibited at each specific concentration of antimicrobial used, given that these bacteria have survived up to that concentration. In addition, statistics such as the MIC_50_ (median MIC) and MIC_90_ (90^th^ percentile MIC) are readily visible. The drawback of a traditional survival approach is that the proportional hazard and the continuous time data assumptions are typically not met. Instead, we analyzed our MIC data using a logistic model adapted for discrete-time survival data [33], [51], [52], [53]. Discrete time in our analysis was analogous to the specific concentrations (dilutions) of the antimicrobials on a log_2_-transformed scale (plus four to avoid negative values). The recorded event was the inhibition of the bacterial growth at an observed minimum concentration.

Fitted survival curves from the discrete-time regression model indicated that administration of both CTC and CCFA selected for higher proportions of isolates that could grow (i.e., were not inhibited) at higher *in vitro* ceftiofur concentrations. The CCFA treatment effect on MIC distributions was much more profound than the CTC treatment effect. Pens in which all animals received both CCFA and CTC treatments had 26% of the isolates that were still able to grow at the highest ceftiofur concentration on the NARMS panel (right-censored on survival curve, MIC above the highest concentration used (8 µg/ml)). Meanwhile, isolates obtained from pens in which only one animal received CCFA treatment, and without subsequent CTC treatment, had almost all of their isolates inhibited by the highest ceftiofur concentration. Differences also emerged among the treatment groups at much lower ceftiofur concentrations (see Figure 9: 2 and 3 on the X-axis, corresponding to concentrations of 0.25 and 0.5 µg/ml, respectively). These curves provide useful information and permit direct comparison of overall trends of the MIC distribution over the entire antimicrobial dilution range for all four groups, rather than simply comparing the proportion resistant/susceptible among the four treatment groups.

In conclusion, CTC treatment resulted in an increased probability of recovering ceftiofur resistant isolates both at phenotypic and genotypic levels. Chlortetracycline appears to greatly exacerbate ceftiofur resistance levels following CCFA therapy and therefore should be avoided, especially when used in sequence. Unsurprisingly, pen-level ceftiofur resistance was lower in the groups with individual CCFA-treated and other non-treated animals co-housed. Further studies are required to establish the effects on the levels of antimicrobial resistance in individual animals of co-housing antimicrobial-treated and non-treated animals at these and other varying ratios. Such information will assist in determining some of the risks/benefits of individual- versus mass-therapy in production agriculture settings.
